# Homeopathy for allergic rhinitis: protocol for a systematic review

**DOI:** 10.1186/2046-4053-3-59

**Published:** 2014-06-10

**Authors:** Kushal Banerjee, Ceire Costelloe, Robert T Mathie, Jeremy Howick

**Affiliations:** 1 Dr. Kalyan Banerjee’s Clinic, I-1691 Chittaranjan Park, New Delhi 110019, India; 2 Centre for Primary Care and Public Health, Blizard Institute, Yvonne Carter Building, 58 Turner Street, London E1 2AB, UK; 3 British Homeopathic Association and Faculty of Homeopathy, Hahnemann House, 29 Park Street West, Luton, Bedfordshire LU1 3BE, UK; 4 Centre for Evidence-Based Medicine, Department of Primary Care Health Sciences University of Oxford, New Radcliffe House, 2nd floor, Jericho, Oxford OX2 6NW, UK

**Keywords:** Homeopathy, Homeopathic, Allergic, Allergy, Rhinitis, Hay fever, Pollinosis, Rhinorrhea, Hypersensitivity

## Abstract

**Background:**

Allergic rhinitis is a global health problem that is often treated with homeopathy. The objective of this review will be to evaluate the effectiveness of homeopathic treatment of allergic rhinitis.

**Methods/Design:**

The authors will conduct a systematic review. We will search Medline, CENTRAL, CINAHL, EMBASE, AMED, CAM-Quest, Google Scholar and reference lists of identified studies up to December 2013.

The review will include randomized controlled trials that evaluate homeopathic treatment of allergic rhinitis. Studies with participants of all ages, with acute or chronic comorbidities will be included. Patients with immunodeficiency will not be included. The diagnosis will be based on the published guidelines of diagnosis and classification. Studies of all homeopathy modalities (clinical, complex and classical homeopathy, and isopathy) will be included. We will include trials with both active controls (conventional therapy, standard care) and placebo controls.

The primary outcomes are: an improvement of global symptoms recorded in validated daily or weekly diaries and any scores from validated visual analogue scales; the total Quality of Life Score (such as the Juniper RQLQ);individual symptoms scores which include any appropriate measures of nasal obstruction, runny nose, sneezing, itching, and eye symptoms; and number of days requiring medication. Secondary outcomes selected will include serum immunoglobin E (IgE) levels, individual ocular symptoms, adverse events, and the use of rescue medication.

Treatment effects will be measured by calculating the mean difference and the standardized mean difference with 95% confidence interval (CI) for continuous data. Risk ratio or, if feasible, odds ratio will be calculated with 95% CI for dichotomous data. After assessing clinical and statistical heterogeneity, meta-analysis will be performed, if appropriate. The individual participant will be the unit of analysis. Descriptive information on missing data will be included about participants missing due to drop out, whether there was intention to treat or per protocol analysis and missing statistics. A number of subgroups, homeopathic potency, age groups, and types of allergic rhinitis (seasonal or perennial) will be analyzed. Sensitivity analysis will be performed to explore the impact of risk of bias on overall treatment effect.

**Systematic review registration:**

PROSPERO CRD42013006741

## Background

### Description of the condition

Rhinitis is described as inflammation of the nasal mucosa and includes common symptoms like nasal discharge, itching, sneezing, nasal blockage, or congestion. There are three types of rhinitis commonly seen in clinical practice: allergic, non-allergic, and infective. Mixed forms also occur [1]. Allergic rhinitis (AR) is an immunoglobin E (IgE)-mediated immunologic response of the nasal mucosa to airborne allergens such as pollens, dust, or animal dander. Inhalation of allergens in individuals with a sensitized immune system produces degranulation of mast cells with the release of chemical mediators. These mediators are responsible for the symptoms of AR. AR is clinically defined by the presence of rhinorrhea, nasal obstruction, nasal itching, and sneezing which are reversible spontaneously or with treatment [2]. Rhinitis affects quality of life, performance and attendance at school [3], and work. It has significant impact on healthcare costs [4]. Allergies are responsible for an estimated annual expenditure of one billion pounds in the National Health Services in the United Kingdom [5]. Conventional treatment includes oral or topical antihistamines, intranasal or systemic corticosteroids, and allergen immunotherapy. A cohort of 16-year-olds in the United Kingdom showed 23% to be suffering from hay fever in 1986 [6].

AR is also a global health problem, affecting 500 million patients worldwide (10 to 20% of the population) [7]. It may commonly be caused by house dust mites, pollen from grass, trees and weeds, and animal dander. AR can be broadly classified into seasonal and perennial. AR has been found to significantly impair psychological wellbeing and perceived to impair cognitive functioning [8]. The prevalence of AR is also high in developing nations: rhino-conjunctivitis was 15.3% amongst 11 to 15 year old school-going children in northern Africa [9]. In addition, there is growing evidence to support a link between allergic rhinitis and asthma [10].

Several trials have evaluated the effectiveness of homeopathy for AR. Results from these trials are mixed and their quality is poor. One systematic review involving a single homeopathic remedy (*Galphimia glauca*) for treating AR has been published in English [11]. Three out of four studies included in that review reported significant results in favor of the intervention, however, it only included one homeopathic medicine. Two important drawbacks of this review are that of the included trials, neither used validated outcome measures nor intention-to-treat analyses. Passalacqua *et al*. [12] conducted a systematic review on complementary and alternative medicine for rhinitis and asthma, concluding that the evidence for a specific effect of homeopathy is weak. Bellavite *et al*. [13] conducted a descriptive review of clinical research on advances in homeopathy and immunology which included AR. No meta-analysis was performed in this review. Apart from Ernst [11], no systematic review evaluating only homeopathic treatment for allergic rhinitis has been published in English.

### Why is it important to do this review?

No systematic review of all homeopathy modalities for treating AR has been conducted. A problem with evaluating classical homeopathy is that it involves an extensive consultation and an individualized treatment regimen. Treatment regimens are decided based on disease state and information gathered in the comprehensive homeopathic case-taking by a qualified homeopathic physician. Hence two patients with the same (conventional) diagnosis may receive very different homeopathic treatments. Classical homeopathy therefore involves more extensive consultations which themselves might have effects, at least for subjective outcomes. Hence apparent positive effects of classical homeopathy might be difficult to disentangle from the effects of longer consultations and other ‘context effects’ [14,15]. Clinical homeopathy, on the other hand, is delivered in much the same way that conventional drugs are prescribed. Complex homeopathy is similar to clinical homeopathy but more than one remedy is prescribed in a single formulation. Neither clinical nor complex homeopathy requires lengthy and comprehensive homeopathic case-taking [16,17]. In both of these forms the intervention is linked to a disease state and not individualized for every patient. Clinical homeopathy, isopathy, and complex homeopathy could also be more cost-effective and therefore more feasible in environments with limited resources such as India.

A systematic review that includes clinical, complex homeopathy and isopathy is therefore required to assess the benefits of homeopathy for treating AR.

## Methods/Design

### Description of the intervention

The review will include trials of clinical, complex, isopathic, and classical homeopathy. Homeopathic remedies are defined as medicines listed in homeopathic *materia medica* (texts containing symptoms of homeopathic remedies) and prepared according to the homeopathic pharmacopoeia of the country in which the trial is being conducted.

### Objective

Our primary objective is to determine the efficacy and effectiveness of homeopathic treatment of AR. Our secondary aim is to compare the effectiveness of different forms of homeopathy on AR.

### Criteria for including studies for this review

#### ***Types of studies***

Randomized, double-blinded (both patients and doctors blinded), controlled trials comparing homeopathy (classical, clinical, isopathic, or complex) with conventional treatment, other homeopathy, or placebo, for the treatment of seasonal or perennial AR in patients of any age.

#### ***Types of participants***

All age groups (newborn to adult) suffering from any form of AR will be included. These may include participants with acute or chronic comorbidities but without immunodeficiency. Participants may also include those on conventional treatment for other health issues. Symptoms of allergic rhinitis include rhinorrhea, nasal obstruction, nasal itching, and sneezing which are reversible spontaneously or with treatment [2]. A diagnosis of AR will include the following symptoms based on the Guideline Summary of Management of Allergic and Non Allergic Rhinitis [7]:

Nose: nasal discharge (runny nose),sneezing, nasal blockage or congestion, and itchy nose and palate. Eye: bilateral itchy eyes, red eyes (concomitant allergic conjunctivitis), and swollen eyes.

There is no specific duration of symptoms required for diagnosis; however, a very short or non-repetitive history of symptoms generally excludes a diagnosis of AR. The presence of any one of the nasal symptoms will be essential for a diagnosis of AR except when nasal blockage is the only symptom. Nasal blockage on its own rarely indicates allergy [2]. The duration of symptoms will not be present as a criterion since this has not been defined for a diagnosis of AR.

### Exclusion criteria for systematic review

The exclusion criteria for this review are as follows: conference proceedings and other abstracted articles in which risk-of-bias assessments cannot usefully be applied; studies in which a non-randomized method of sequence generation and/or a single-blinded approach is explicitly used by the authors; studies using formulations not described by the authors as ‘homeopathic’; studies with a crossover trial design; and studies in which homeopathy is combined with another intervention.

The exclusion criteria for quantitative data extraction (for meta-analysis are: studies from which no data are provided or data are otherwise not extractable. The latter category includes studies with continuous outcomes from which standard deviations are not derivable and those that report non-parametric data only.

#### ***Investigations***

Investigations are not always required to confirm diagnosis. However, a confirmation of allergic rhinitis sometimes involves a skin prick test and/or specific and serum total IgE tests [18]. The use of investigations for confirming diagnosis will not be an inclusion criterion. Trials allowing the administration of conventional treatment as a rescue medication will be included.

#### ***Types of interventions***

We will include trials involving experimental homeopathic treatments delivered orally, through olfaction, or applied on the body. Therefore, globules, nasal sprays, ointments, and other applications prepared with homeopathic medicines will be included in this review. In these trials the homeopathic preparations may contain a single medicine or more than one medicine as in complex homeopathy, the homeopathic interventions may be administered as one single preparation or more than one preparation, and the comparators in the included trials will be placebos or conventional treatment which may include antihistamines, immunotherapy, and decongestants and so forth.

The alleged difference between homeopathy type (classical, clinical, and complex) is important and might be inadequately reported. To deal with this we will use two strategies. First, we will examine individual studies to verify the classification of homeopathy type (classical, clinical, complex, or isopathy) using standard definitions [16]. These are:

‘Classical [individualized] homeopathy: When a single homeopathic remedy was selected based on the total symptom picture of a patient.

Clinical homeopathy: When one or several single remedies were administered for standard clinical situations or conventional diagnoses.

Complex homeopathy: When multiple remedies were mixed into a standard formula to ‘cover’ a person’s symptoms and diagnoses.

Isopathy: When serial agitated dilutions were made from the causative agent in an infectious or toxicological condition’ (from Linde *et al*. [16]).

Therefore when classical homeopathy is used, any homeopathic medicine may be selected based on comprehensive case taking. In clinical homeopathy, for example, *Allium cepa* (a common homeopathic remedy from onions) may be prescribed based on indications that match the clinical picture of allergic rhinitis. In complex homeopathy a preparation containing *Luffa operculata*, *Galphimia glauca*, *Histamine* and *Sulphur*[19] may be used to treat AR.

A preparation from an allergen such as *Betula* (birch) [20] is an example of an isopathic intervention for AR. In practice, medicines used by clinical homeopaths may also be prescribed by classical homeopaths should they be indicated after comprehensive case-taking. By classifying included studies according to type of homeopathy, this review will inform practitioners for the first time about homeopathic medicines according to homeopathy type for AR.

The current authors’ classification, made according to these published definitions, will override reported classifications where appropriate. Second, we will conduct a subgroup analysis to detect any differences between homeopathy types for any outcome measure. If a particular type fails to demonstrate a statistically significant benefit over another then the debate over homeopathy types becomes moot. On the other hand, if we do find a statistically significant difference in effects then we will have shown the importance of specifying homeopathy type. There will be no limit on the maximum dilution of the homeopathic medicine. Trials with homeopathic ‘mother tinctures’ will be included in this review as part of subgroup analyses (see below).

#### ***Primary outcomes***

The primary outcomes of this review are as follows: the improvement of global symptoms recorded in validated daily or weekly diaries and any scores from validated visual analogue scales; the total Quality of Life Score (such as the Juniper RQLQ); individual symptoms scores which include any appropriate measures of nasal obstruction, runny nose, sneezing, itching, and eye symptoms; and the number of days requiring medication. In case of child participants, AR symptoms rated by parents will be considered acceptable.

#### ***Secondary outcomes***

The secondary outcomes of this review are as follows: IgE levels, individual ocular symptoms, any adverse event, hospitalization due to an adverse event, and use of conventional medication (frequency and quantity).

### Search

#### ***Electronic search***

The search to locate randomized trials will be based on the Cochrane Highly Sensitive Search Strategy [21]. Manual search of the results will supplement the electronic search.

Medline (1946 to December 2013 inclusive) on Ovid, CENTRAL, The Cochrane Ear Nose and Throat Disorder Group Trials Register, CINAHL, EMBASE (1974 to December 2013) on Ovid, Allied and Complementary Medicine Database (AMED) (1985 to December 2013) on Ovid, CAM-Quest and Google Scholar.

The search will not have any language filters. The reference lists of identified studies will be searched for additional trials and trial authors will be contacted if data are missing from reports of studies. Filters for randomized controlled trials, human trials, and so forth will not be applied.

The search strategy that will be used to search Medline and adapted for the other sources is detailed in Table 1.

**Table 1:** Search strategy: homeopathy for allergic rhinitis

**Step**	**Search procedure**
1	Homeopathy/
2	(Homoeopathy or homoeopathic or homeopathic).mp. [mp = title, abstract, original title, name of substance word, subject heading word, keyword heading word, protocol supplementary concept, rare disease supplementary concept, unique identifier]
3	1 or 2
4	Rhinitis, Allergic, Perennial/or Rhinitis, Vasomotor/or Rhinitis/or Rhinitis, Atrophic/or Rhinitis, Allergic, Seasonal/
5	(nasal congestion or rhinorrhea or rhinorrhea or sneezing or itchy nose).mp. [mp = title, abstract, original title, name of substance word, subject heading word, keyword heading word, protocol supplementary concept, rare disease supplementary concept, unique identifier]
6	Exp Rhinitis/
7	(rhiniti* or rhinoconjunctivitis or SAR or PAR).mp. [mp = title, abstract, original title, name of substance word, subject heading word, keyword heading word, protocol supplementary concept, rare disease supplementary concept, unique identifier]
8	(hay fever or hay next fever or pollinosis or pollenosis).mp. [mp = title, abstract, original title, name of substance word, subject heading word, keyword heading word, protocol supplementary concept, rare disease supplementary concept, unique identifier]
9	4 or 5 or 6 or 7 or 8
10	3 and 9

Bibliographies of identified studies and reviews will be hand searched.

### Selection of studies

Studies will be eligible for inclusion if they have a randomized, blinded and controlled design, validated measures of outcome, and evaluate homeopathic treatment for AR.

Two review authors (KB and JH) will independently review titles and abstracts to select potentially eligible studies. This will be followed by a full text analysis of the selected studies to assess compliance with the eligibility criteria. Disagreements will be resolved by discussion first. A third author (CC) will arbitrate if the disagreement is due to a difference in interpretation. If no clear categorization can be made of a selected study it will be categorized as one that is awaiting assessment. Study authors will be contacted for further information wherever necessary. RTM may be asked to categorize the study. The selected studies will then be further evaluated for methodological quality (risk of bias).

### Data extraction and management

A data extraction form will be designed and the relevant details will be transferred to the standard data extraction sheet after the form is reviewed by all authors. This will cover study type and methods including number and description of participants; details of type, mode of administration, dosage, and duration of intervention; and type, timing, and measurement method of outcomes. The authors, publication year, and journal of publication will also be recorded. Two review authors (KB and RTM) will independently extract data from every included trial to minimize error and reduce potential bias.

### Assessment of risk of bias in included studies

Two review authors (KB and JH) will independently assess the risk of bias of each study using the Cochrane Risk of Bias Tool from the Cochrane Handbook for Systematic Reviews of Intervention [22]. These consist of sequence generation, allocation concealment, blinding of participants and personnel, blinding of outcome assessment, incomplete outcome data, selective outcome reporting, and other sources of bias.

Each of these factors will be described as reported in the trial. Attributes of ‘low’, ‘high’, or ‘unclear’ risk will be made for each of them. We will resolve any disagreements about inclusion of a study by discussion and consensus first. A third reviewer (CC or RTM) will arbitrate if disagreement is due to difference in interpretation. Trial authors will be contacted if clarification is required.

### Measurement of treatment effect

Baseline comparability of treatment groups for known prognostic variables will be assessed. For continuous data, individual and pooled statistics as mean difference and standardized mean difference with 95% CI will be calculated. In case of dichotomous outcomes, risk ratios (or, if feasible, odds ratios) will be calculated with 95% CI. If appropriate, meta-analysis will be performed after assessment of clinical or statistical heterogeneity. The individual participant will be the unit of analysis. Descriptive information on missing data will be included about participants missing due to dropout, whether there was intention-to-treat or per-protocol analysis, and about missing statistics.

### Assessment of heterogeneity and reporting biases

Heterogeneity will be assessed by comparing trial populations, settings, and methods. Statistical heterogeneity will be assessed using the I^2^ statistic. A value of greater than 50% will be considered as important heterogeneity. Both fixed-effect and random-effect models will be applied to the data. If the fixed effect and random effect meta-analyses give identical results then it is unlikely that there is important statistical heterogeneity, and the fixed-effect result will be presented. If the results vary, possible causes of heterogeneity will be examined. This will inform which result should be reported - stable robust techniques with an underlying assumption of a fixed effect (which may be incorrect) or less stable, sometimes unpredictable techniques based on an underlying assumption of random effect (which may be more likely). A sensitivity analyses will be performed by conducting the meta-analysis including and excluding studies which were deemed to affect heterogeneity. Changes, if any, will be reported in a table. Funnel plots will be constructed, if possible. This is a scatter plot of the intervention effect estimates from individual studies against some measure (usually the standard error of the mean) of each study’s sample size or precision.

### Data synthesis

Review Manager (RevMan) [Computer program], Version 5.2. Copenhagen: The Nordic Cochrane Centre, The Cochrane Collaboration, 2012. RevMan 5.2 will be used for data synthesis and meta-analysis. The primary analyses are homeopathy (all forms) compared with placebo, and homeopathy (all forms) compared with conventional treatment. The additional analyses are: (1) clinical homeopathy compared with placebo; (2) clinical homeopathy compared with conventional treatment; (3) complex homeopathy compared with placebo; (4) complex homeopathy compared with conventional treatment; (5) classical homeopathy compared with placebos; and (6) classical homeopathy compared with conventional treatment. We shall compare the pooled effect sizes of: #1 and #3 and #5; #2 and #4 and #6.

### Subgroup analyses

If sufficient data are available, subgroup analyses will be conducted to investigate the effect of homeopathic potency (mother tincture; potency <12C; potency >12C); different age groups (children versus adults) and different types of AR (perennial versus seasonal). It is recognized that there will be relatively low power to detect effects at the subgroup level; hence these analyses will be undertaken with the aim of hypothesis generation only.

### Sensitivity analyses

A sensitivity analysis will be performed to explore the impact of risk-of-bias on overall treatment effect. Studies will be pooled according to low, uncertain or high risk of bias. The contribution of studies to heterogeneity will be assessed.

## Discussion

This will be the first systematic review of all types of homeopathy for allergic rhinitis. We have attempted to design a robust protocol which should result in an objective and adequate summary of the available evidence in this area of research. The review is not limited to studies published in the English language: such limitation might have excluded several studies that met all other inclusion criteria. The selected databases index a large number of CAM journals and the search strategy is expected to identify most of the relevant studies that exist.

## Abbreviations

AMED: Allied and Complementary Medicine Database; AR: Allergic Rhinitis; CAM: Complementary and Alternative Medicine; IgE: Immunoglobulin E.

## Competing interests

The authors declare that there are no competing interests.

## Authors’ contributions

KB conceived of the review, designed, and wrote the protocol. KB will carry out the searches, extract data, conduct risk-of-bias assessment, carry out the meta-analysis, draw the conclusions, and write the manuscript for the review. JH edited the protocol, will carry out the searches, extract data, conduct risk-of-bias assessment, will draw conclusions, and edit the manuscript for the review. CC and RTM edited the protocol, will resolve issues relating to inclusion of studies, risk-of-bias assessment, and conclusions. CC and RTM will assist KB with meta-analysis and edit the manuscript. All authors have approved the protocol. All authors read and approved the final manuscript.

## Authors’ information

KB is a homeopath practicing in New Delhi, India and has completed the coursework for a Master of Science in Evidence Based Healthcare from the University of Oxford. JH is a senior research associate at the University of Oxford.CC is lecturer in medical statistics at the Clinical Trials Unit at the Queen Mary University of London.RTM is the Research Development Adviser of the British Homeopathic Association, UK.
